# Estimating the adverse selection and moral hazard in Urban and Rural Resident Basic Medical Insurance of China: a semi-parametric estimation approach

**DOI:** 10.3389/fpubh.2025.1723767

**Published:** 2026-01-07

**Authors:** Yue Wu, Jiaxi Xu

**Affiliations:** School of Insurance, Central University of Finance and Economics, Beijing, China

**Keywords:** adverse selection, kernel density estimation, moment estimation, moral hazard, Urban and Rural Resident Basic Medical Insurance

## Abstract

**Introduction:**

This study investigated the causes of excessive medical resources consumption, by disentangling and estimating the adverse selection and moral hazard within China’s Urban and Rural Resident Basic Medical Insurance (URRBMI), with the aim of proposing targeted solutions to this issue.

**Methods:**

The analysis employed a utility optimization model of residents’ medical consumption. Parameters were estimated via kernel density estimation, moment estimation, and bootstrap methods, using data from the China Family Panel Studies (32,822 observations across 31 provinces for 2020 and 2022). Adverse selection was assessed by comparing the health distributions of the insured and uninsured derived from the model, while moral hazard was examined by constructing a counterfactual scenario based on Slutsky decomposition within the modeling framework.

**Results:**

The results indicate that the insured had poorer average health status than the uninsured in both 2020 and 2022, demonstrating adverse selection in URRBMI. This issue intensified by 2022 following a premium increase. Moral hazard was also identified, particularly among the older population, higher-income, less healthy, and more educated enrollees. Specifically, the moral hazard incidence rate was 46.63% in 2020, leading to per capita medical over-consumption of 204.57 yuan, accounting for 13.8% of total medical expenditures. Following the implementation of DRG/DIP payment reform, the over-consumption decreased in 2022 to 187.63 yuan, with the proportion falling to 10.6%.

**Discussion:**

To address the excessive medical resources consumption, policymakers should focus on two key measures: expanding insurance coverage while increasing government premium subsidies to mitigate adverse selection, and advancing payment system reforms to curb the moral hazard induced medical over-consumption.

## 1 Introduction

In recent years, China has actively pursued reforms to its medical insurance system in an effort to enhance the overall health of the population and improve accessibility to healthcare services. Since 2016, the government has integrated the New Cooperative Medical Scheme (NCMS) and the Urban Resident Basic Medical Insurance (URBMI) into Urban and Rural Resident Basic Medical Insurance (URRBMI) to improve the benefits of the basic medical insurance (1). According to the Statistical Bulletin of China’s Health and Health Development, China’s total healthcare expenditure reached 9,057.58 billion yuan in 2023, of which personal healthcare expenditure accounted for 27.3%, down 15.61% from 2016. And the average growth rate of personal healthcare expenditure over the past decade was 10.69%, which was lower than the 13.63% growth rate of total healthcare expenditure. It is evident that the reform has alleviated the healthcare burden on residents to some extent. However, the continuous expansion of basic medical insurance coverage and enhancement of benefits have also spurred an increase in total healthcare expenditure, particularly in government and social healthcare expenditure, thereby generating funding pressures within the medical insurance system. Fundamentally, the exacerbation of information asymmetry issues among medical insurance administrative departments, insured residents, and the healthcare providers serves as the primary driver of the rapid growth in total healthcare expenditure.

Specifically, the consequences arising from information asymmetry are adverse selection and moral hazard (2). China’s URRBMI is a non-compulsory public insurance scheme, and its premium is set according to the regional economic levels and participants’ type, such as students, the older adult, and flexible workers. Such a design, however, may lead to adverse selection, as residents in poorer health are more likely to be insured. Additionally, the moral hazard arises when the insured are more likely to consume healthcare services than the uninsured, divided into ex-ante MH (neglecting health management after enrollment) and ex-post MH (increased healthcare demand due to insurance coverage). Obviously, both adverse selection and moral hazard would lead to excessive growth in healthcare expenditure and misallocation of medical resources. However, addressing the problems caused by adverse selection or moral hazard often exacerbates the market inefficiency caused by the other. Therefore, investigating the issue of information asymmetry, particularly by integrating adverse selection and moral hazard into a unified analytical framework for assessment holds a substantial significance.

For a long time, the study of information asymmetry has been a significant research topic in health economics. Arrow (2) was the first to point out that information asymmetry between the supply and demand sides of healthcare services, as well as with insurance providers, would lead to adverse selection and moral hazard, thereby causing the excessive medical resources consumption and market inefficiency. Subsequently, numerous scholars have embarked on empirical analyses to determine the existence and extent of adverse selection and moral hazard in the health insurance market (3–5). The central idea of identifying adverse selection is to test whether there is a positive correlation between insurance coverage and risk level (6–9). Chiappori and Salanie (10) firstly proposed a positive correlation test based on a two-sided discrete choice model and estimated the degree of insurance coverage and risk level separately. Adverse selection was then identified by examining whether the error terms of the two models exhibited positive correlation. However, the examination of moral hazard is often intertwined with adverse selection. This is because a positive correlation between enrollment and healthcare utilization cannot provide sufficient evidence of moral hazard, for the variable of enrollment can no longer satisfy the exogeneity requirement in the presence of adverse selection (11–13). Even without adverse selection, attributing the rise in enrollees’ medical expenditure to moral hazard is still biased, since the release of normal medical demand due to enrollment can also lead to an overestimation of moral hazard (14–16). Therefore, integrating adverse selection and moral hazard into a unified framework for analysis and identification has become a focal point in the empirical study of information asymmetry.

The commonly employed identification methods in the existing literature can be categorized into three types. The first is randomized or natural experiment method. The fundamental concept of the randomized experiment method involves researchers artificially creating exogenous shocks and observing the impact of these shocks on the behavior of the insured. A representative example is the field-controlled health insurance experiment conducted by the RAND corporation in the United States from 1974 to 1982, commonly referred to as the “RAND Experiment.” In this experiment, the participants were randomly divided into two groups and offered health insurance with different coverage to different groups (17). Ultimately, it was found that group members offered higher coverage had significantly higher medical consumption than other groups. Since the samples were randomly grouped, eliminating the influence of adverse selection, the results of the experiment confirmed the existence of moral hazard (18).

Because randomized experiments consume considerable labor and material resources, scholars have begun to make more use of natural experiments (19–22). The natural experiment approach primarily identifies adverse selection and moral hazard based on exogenous shocks resulting from changes in insurance policies. For instance, Chiappori et al. (19) tested moral hazard using a policy change in 1994, in which French social health insurance changed from full benefit to co-payment. Similarly, Culter and Reber (20) detected adverse selection by studying the rollout of a new employee health insurance plan at Harvard University. In the Chinese context, Zhao et al. (21) exploited a natural experiment arising from the integration of urban and rural medical insurance in Chengdu. After controlling for adverse selection, their findings revealed the evidence of moral hazard. Complementing this, Xiang et al. (22) employed a quasi-experiment derived from differential adjustments in reimbursement policies during the transition from county-level to municipal-level insurance coordination in two adjacent counties. Using 2008–2013 panel data on hospitalization expenditures, they obtained an estimate of moral hazard after netting out the influence of adverse selection.

The second approach is based on the characteristics of insurance market, which were first used in the automobile insurance market (23). Cohen (23) found that the positive correlation between enrollment and indemnity differed significantly among different driving age groups. Novice drivers can be considered without adverse selection because of their low mastery of driving risks; therefore, the positive correlation between enrollment and indemnity that occurs in the higher driving age group can be used as evidence of adverse selection. For health insurance, some scholars have also used local health insurance characteristics to distinguish between adverse selection and moral hazard (24–28). Nguyen (27) analyzed differences between compulsory and voluntary insurance participants in Vietnam, estimating moral hazard through medical consumption disparities and identifying adverse selection by comparing insured characteristics across both insurance types. Similarly, Feng et al. (28) utilized data from the China Family Panel Studies (CFPS) and leveraged the unique characteristic in China where flexible employment groups can voluntarily enroll in the Urban Employee Basic Medical Insurance (UEBMI). By empirically disentangling the adverse selection effect from moral hazard, their study verified that flexible workers who chose to participate in the UEBMI were those with higher medical expenses, thereby confirming the presence of adverse selection.

The third method mainly uses the dynamic panel data, which was first proposed by Abbring et al. (29) in the automobile insurance market, and was theoretically analyzed for its feasibility (30). In the study of health insurance, numerous scholars have employed dynamic panel data to analyze adverse selection and moral hazard (31–37). Dong (34) developed structural equations using panel data from the U. S. Health and Retirement Study to empirically examine the causal relationships between health insurance status, health behaviors, and medical utilization, effectively distinguishing adverse selection from moral hazard. Similarly, Bardey and Buitrago (35) established the presence of adverse selection by demonstrating a positive correlation between prior medical consumption and insurance enrollment, thereby ruling out moral hazard. In the Chinese research, Yuan et al. (36) used panel data from the CHNS database from 1989 to 2009 to estimate adverse selection and moral hazard using the logit model and fixed effects model, respectively. Yin and Liu (37) also dynamically distinguished and tested moral hazard and selection effects in commercial health insurance by analyzing the relationship between current enrollment choice and previous health status.

In summary, empirical studies on information asymmetry have focused on obtaining accurate assessments of adverse selection and moral hazard by excluding the effects of other factors. However, the commonly used methods all face certain limitations, such as requirements for policy shock, market characteristics, or data continuity. Departing from the aforementioned approaches, our study based on the foundational model introduced by Bajari et al. (38), employing a unified framework to investigate the adverse selection and moral hazard within the URRBMI. While Zhong et al. (39) applied the same model to estimate residents’ latent health status and assess adverse selection without extending to moral hazard, Wang and Zhu (40) only focused on estimating moral hazard by constructing counterfactual scenario when uninsured residents transition to being insured based on Bajari et al. (38). Our study advances this research by estimating both adverse selection and moral hazard-the latter through constructing counterfactual scenario based on Slutsky decomposition. Furthermore, by leveraging the natural experiment of China’s medical insurance payment reform since 2022, we identify the causal effect of the reform and moral hazard. These findings provide a scientific basis for proposing targeted solutions to address the issue of excessive medical resources consumption, and refining China’s medical insurance system.

## 2 Materials and methods

### 2.1 Background

The Urban and Rural Residents Basic Medical Insurance is a social insurance program established by the Chinese government to fulfill the basic healthcare needs of urban and rural populations. It primarily aims to cover those not insured under the Urban Employee Basic Medical Insurance (UEBMI), including children, students, older adults, the unemployed, and other non-working residents. The premium levels are determined according to regional economic development, varying across localities and time periods. Financing is structured through a combination of individual contributions and government subsidies, with the latter covering a major portion to ensure affordability and broad accessibility. Reimbursement rates, meanwhile, are contingent upon the healthcare services type and the tier of the healthcare facility. Generally, inpatient services generally enjoy higher reimbursement rates than outpatient care, and higher-level hospitals tend to apply lower reimbursement rates compared to primary-level institutions.

Taking city S, a northern provincial capital, as an example, the annual individual premiums in 2022 were 300 yuan for children, 240 yuan for university students, and 360 yuan for adult residents, respectively. And the reimbursement rules for the 2022 URRBMI in city S are as shown in Table 1. Firstly, the general outpatient expenditure refers to medical expenses incurred by insured residents for common outpatient illnesses covered by the medical insurance catalog at primary hospitals. Under city S’s 2022 URRBMI, the reimbursement rate for general outpatient in primary hospitals is 50%, with no deductible but a reimbursement cap of 500 yuan. And the visits for general outpatient services at secondary and tertiary hospitals are not be covered by the URRBMI in city S. However, patients with certain chronic or special diseases (e.g., malignant tumors, uremic dialysis, coronary heart disease) often require long-term outpatient treatment with substantial associated costs. To alleviate the financial burden imposed by managing such conditions and prevent unnecessary hospitalization, the URRBMI has established dedicated reimbursement protocols. Based on the 2022 regulations in city S, 21 chronic and special diseases were designated for enhanced outpatient coverage. Patients receiving treatment for these conditions at primary, secondary, and tertiary hospitals are eligible for reimbursement rates of 80, 70, and 60%, respectively. Additionally, the annual reimbursement cap for outpatient of chronic or special diseases is combined with inpatient reimbursement, totaling 250,000 yuan. Finally, as indicated in Table 1, all inpatient reimbursements are subject to a deductible, which increases with higher hospital tiers. And the reimbursement rate decreases as the hospital level increases, set at 80, 70, and 60% for primary, secondary, and tertiary hospitals respectively, with a unified reimbursement cap of 250,000 yuan.

**Table 1:** Reimbursement rules of URRBMI in City S for 2022.

	Deductible			Reimbursement rate			Reimbursement cap
	Primary hospital	Secondary hospital	Tertiary hospital	Primary hospital	Secondary hospital	Tertiary hospital	
General outpatient	0	–	–	50%	–	–	500
Outpatient for chronic and special diseases	0	200	200	80%	70%	60%	250,000
Inpatient	400	400	1,000	80%	70%	60%	250,000

The units of deductible and reimbursement cap are yuan; Primary hospital serves as primary healthcare institutions, offering integrated medical, preventive, rehabilitative, and general healthcare services to local communities. Secondary hospital acts as regional healthcare centers, providing services to multiple communities. Tertiary hospital, the highest tier, provides healthcare services at the regional, provincial and even national levels.

### 2.2 Database

Our study employs the 2020 and 2022 China Family Panel Studies (CFPS) database for its empirical analysis. Launched in 2010 by the Institute of Social Science Survey at Peking University, the CFPS project is designed to conduct longitudinal tracking surveys of Chinese households. The initial sample encompassed 16,000 households across 25 provinces in China, with the survey scope progressively expanding over time. The database provides extensive individual-level information, including gender, age, income, as well as the insurance status, total outpatient and inpatient medical expenses, insurance reimbursement, and out-of-pocket payments during the survey years. With its broad sampling scope and comprehensive coverage, the database is highly suitable for supporting our study. To investigate the information asymmetry in URRBMI, we exclude residents enrolled in the UEBMI and the supplement medical insurance. This process yields 17,027 valid observations for 2020 and 15,795 for 2022. Descriptive statistics for the sample are summarized in Table 2.

**Table 2:** Descriptive statistics.

	Variables	2020				2022			
		Min	Max	Mean	*N*	Min	Max	Mean	*N*
The insured	Outpatient medical expenses	2	100,000	1,886.256	7,958	1	60,000	2,146.651	7,901
	Outpatient out-of-pocket	0.000	5,500	1,502.929	7,958	0	60,000	1,800.512	7,901
	Outpatient reimbursement rates	0.002	1.000	0.494	1,911	0.003	1.000	0.468	1,838
	Inpatient medical expenses	50	72,000	7,977.377	1,158	100	90,000	8,779.833	1,291
	Inpatient out-of-pocket	0.000	50,000	4,026.286	1,158	0	63,000	4,606.746	1,291
	Inpatient reimbursement rates	0.067	1.000	0.595	918	0.083	1.000	0.578	996
	Income	3,000	5,000,000	31,411.82	14,756	3,000	6,886,000	38,881.34	14,241
	Age	16	92	45.638	14,756	16	97	46.207	14,241
	Gender	0	1	0.501	14,756	0	1	0.494	14,241
	Education	1	4	1.710	14,756	1	4	1.722	14,241
	Marriage	0	1	0.795	14,756	0	1	0.777	14,241
	Children	0	1	0.677	14,756	0	1	0.688	14,241
The uninsured	Outpatient medical expenses	10	100,000	1,338.326	1,147	3	80,000	1,997.383	728
	Inpatient medical expenses	40	180,000	6,441.500	113	500	35,000	7,292.474	39
	Income	3,000	2,011,200	36,941.40	2,271	3,000	3,794,000	53,711.91	1,554
	Age	11	93	39.26	2,271	16	88	39.203	1,554
	Gender	0	1	0.491	2,271	0	1	0.492	1,554
	Education	1	4	1.878	2,271	1	4	1.832	1,554
	Marriage	0	1	0.580	2,271	0	1	0.537	1,554
	Children	0	1	0.474	2,271	0	1	0.472	1,554

The units of medical expenses are yuan; The gender variable is encoded with 0 representing female and 1 representing male; The education level is range from 1 to 4: 1 signifies the completion of primary education, 2 denotes high school graduation, 3 represents the attainment of an undergraduate degree, and 4 indicates the completion of either a master’s or doctoral degree; Marriage is encoded with a binary classification, where 0 denotes unmarried and 1 denotes married; The number of children is coded as 0 for no children and 1 for having children.

In the sample analyzed for our study, the enrollment rate in the URRBMI stood at 86.66% in 2020, which subsequently rose to 90.16% by 2022. Analysis of the 2020 data reveals that a mere 24.01% of residents who sought outpatient services were eligible for reimbursement, with the mean reimbursement rate standing at 49.4%. And a substantial 79.27% of residents who underwent inpatient treatment were reimbursed, with a higher average reimbursement rate of 59.5%. Secondly, compared to the insured, the uninsured exhibit lower probabilities of both outpatient and inpatient visits, and their average medical expenditures were also lower than those of the insured, providing a preliminary indication of adverse selection and moral hazard. In terms of individual characteristics, data from both 2020 and 2022 indicate that the uninsured are, on average, younger and have higher proportions of being unmarried and childless. Given that these residents generally report better health statuses, preliminarily suggesting the presence of adverse selection in URRBMI.

### 2.3 The model

#### 2.3.1 Utility function

Our model relies on the assumption of a rational resident who maximizes his utility function by choosing his optimal healthcare services *m* and consumption of composite goods *c* subject to a budget constraint. Firstly, following the model specification in Bajari et al. (38), we assume that the resident’s utility function is Equation 1:


$$U(c,m;\theta ,\gamma )=F[c,(1-\theta ),\gamma_{1}]+H[m,\theta ,\gamma_{2}]$$ (1)

where $H[m,,\theta ,,\gamma_{2}]$ and $F[c,,(1-\theta ),,\gamma_{1}]$ represent the utility derived from healthcare services *m* and composite goods consumption *c*, which includes all expenditures other than healthcare services. The parameters $\gamma_{1}$ and $\gamma_{2}$ denote the resident’s risk aversion coefficients for composite goods consumption and medical expenditure, respectively. The θ∈[0,1] is interpreted as a proxy for health status and is the resident’s private information. A value of *θ* closer to 1 indicates poorer health, leading to an expectation of higher medical expenditure to improve utility.

The utility function satisfies the following four properties:

(1) $U_{c}>0$ and $U_{m}>0$ , indicating the marginal utilities of composite goods and healthcare services are both positive;
(2) $U_{\mathrm{cc}}<0$ and $U_{\mathrm{mm}}<0$ , implying diminishing marginal utility for both types of consumption;
(3) $U_{\mathrm{cm}}=0$ , assuming that the consumption of composite goods does not affect the marginal utility of medical services, and vice versa;
(4) $U_{c}(0,m,\theta )=\infty$ and $U_{c}(c,0,\theta )=\infty$ , indicating that the marginal utility of either good approaches infinity as its consumption approaches zero.

To theoretically disentangle adverse selection from moral hazard while satisfying the above functional assumptions, we adopt the Equation 2 as individual’s utility function for identification:


$$U(c_{i},m_{i},\theta_{i},\gamma_{1},\gamma_{2})=(1-\theta_{i})\frac{{c_{i}}^{1-\gamma_{1}}}{1-\gamma_{1}}+\theta_{i}\frac{{m_{i}}^{1-\gamma_{2}}}{1-\gamma_{2}}$$ (2)

Firstly, $\theta_{i}$ and $1-\theta_{i}$ indexes the weight that resident *i* places on consumption of healthcare services and composite goods, respectively. The greater the value of $\theta_{i}$ , the higher the medical expenditure required for resident *i* to achieve utility maximization-indicating poorer health status. While $\theta_{i}$ is observed to the resident *i*, it is not observed by the insurer. Therefore, our model provides a method to test for adverse selection by comparing differences in health status distributions between the insured and the uninsured.

Second, the coefficient of resident’s risk aversion is closely related to individual characteristics such as education level, age, and gender, demonstrating certain heterogeneity across different subgroups (41). To account for this heterogeneity, we will select appropriate covariates for grouping and perform group-specific estimation of the coefficient in subsequent analyses. Considering the feasibility of estimation, we assume that the risk aversion coefficients are heterogeneous only across groups but remains constant within each group.

#### 2.3.2 Budget constraint

In our model, consumers face the budget constraint that total spending may not exceed their income. Under the assumption that the market price ratio between composite goods and healthcare services is unity, the effective price of healthcare for an insured resident is determined by the insurance reimbursement rate $a_{i}$ . By contrast, the uninsured pay the full market price, incurring neither a premium nor any reimbursement. Accordingly, the budget constraint faced by resident *i* can be expressed as Equation 3:


$$c_{i}+m_{i}(1-a_{i})\leq y_{i}-p_{i}$$ (3)

where $y_{i}$ is the per capita disposable income of households during an insurance period (1 year in our study); $p_{i}$ is the premium; $a_{i}$ is the actual reimbursement rate, that is, the rate of the actual reimbursement amount to total medical expenses during the insurance period. A difficulty the resident *i* faces is that $a_{i}$ will be uncertain at the time that $m_{i}$ is chosen. This is because medical insurance reimbursement is a specialized process managed entirely by relevant departments, and patients often lack sufficient professional knowledge to fully understand the reimbursement details. Therefore, when making medical consumption decisions, patients can only estimate the actual reimbursement rate based on reimbursement policies and their prior medical experiences.

Through the above analysis, it can be seen that the core of constructing our model lies in characterizing resident’s cognitive process regarding $a_{i}$ . Following Bajari et al. (38), we model $a_{i}$ as a stochastic variable with a conditional probability density function $\;f_{\mathrm{jk}}(a_{i}|m_{i})$ . This specification allows to vary by healthcare service type *j* (outpatient or inpatient) and hospital tier *k*, while also capturing its dependence on medical expenditures.

#### 2.3.3 Hypothesis

In our model, rational residents aim to maximize their utility by making consumption decisions based on their privately observed health status. Considering the randomness of actual reimbursement rate, the resident *i*’s expected utility function can be written as Equation 4, where the subscript *t* indicates the year. The optimal choice $m_{\mathrm{it}}$ chosen by resident *i* in year *t* is determined by a first order condition that $EU_{m}=0$ , as illustrated in Equation 5. By solving Equation 5, the optimal $m_{\mathrm{it}}$ can be obtained. After further processing, we finally obtain the Equation 6 for the resident *i*’s latent health status, where ${\hat{I}}_{\mathrm{it}}$ is a definite integral of $a_{\mathrm{it}}$ , as illustrated in Equation 7.^1^


$$\begin{array}{l} \mathrm{EU}(m_{\mathrm{it}};\theta_{\mathrm{it}},\gamma_{1},\gamma_{2})=\int_{0}^{1}(1-\theta_{\mathrm{it}})\frac{{\left(y_{\mathrm{it}}-p_{\mathrm{it}}-m_{\mathrm{it}}(1-a_{\mathrm{it}})\right)}^{1-\gamma_{1}}}{1-\gamma_{1}}\\ f_{\mathrm{jkt}}(a_{\mathrm{it}}|m_{\mathrm{it}})da_{\mathrm{it}}+\theta_{\mathrm{it}}\frac{m_{\mathrm{it}}^{1-\gamma_{2}}}{1-\gamma_{2}} \end{array}$$ (4)

$$\begin{array}{l} \frac{\partial \mathrm{EU}(\cdot )}{\partial m_{\mathrm{it}}}=\frac{\partial }{\partial m_{\mathrm{it}}}[\begin{array}{l} \int_{0}^{1}(1-\theta_{\mathrm{it}})\frac{{\left(y_{\mathrm{it}}-p_{\mathrm{it}}-m_{\mathrm{it}}(1-a_{\mathrm{it}})\right)}^{1-\gamma_{1}}}{1-\gamma_{1}}\\ f_{\mathrm{jkt}}(a_{\mathrm{it}}|m_{\mathrm{it}})da_{\mathrm{it}} \end{array}]\\ +\frac{\partial }{\partial m_{\mathrm{it}}}(\theta_{\mathrm{it}}\frac{m_{\mathrm{it}}^{1-\gamma_{2}}}{1-\gamma_{2}})=0 \end{array}$$ (5)

$${\hat{\theta }}_{\mathrm{it}}=\frac{{\hat{I}}_{\mathrm{it}}}{{\hat{I}}_{\mathrm{it}}-\frac{\left(1-{\hat{\gamma }}_{1}\right)}{m_{\mathrm{it}}^{{\hat{\gamma }}_{2}}}}$$ (6)

$$\begin{array}{l} {\hat{I}}_{\mathrm{it}}=\int_{0}^{1}[\begin{array}{l} -(1-{\hat{\gamma }}_{1})(1-a_{\mathrm{it}}){\left(y_{\mathrm{it}}-p_{\mathrm{it}}-m_{\mathrm{it}}(1-a_{\mathrm{it}})\right)}^{-{\hat{\gamma }}_{1}}\\ {\hat{f}}_{\mathrm{jkt}}(a_{\mathrm{it}}|m_{\mathrm{it}}) \end{array}]\\ da_{\mathrm{it}}+\int_{0}^{1}[\begin{array}{l} {\left(y_{\mathrm{it}}-p_{\mathrm{it}}-m_{\mathrm{it}}(1-a_{\mathrm{it}})\right)}^{1-{\hat{\gamma }}_{1}}\\ \frac{\partial {\hat{f}}_{\mathrm{jkt}}(a_{\mathrm{it}}|m_{\mathrm{it}})}{\partial m_{\mathrm{it}}} \end{array}]da_{\mathrm{it}} \end{array}$$ (7)

We assume that all individuals recorded in the CFPS are rational economic agents. Consequently, their observed medical expenditures reflect utility-maximizing decisions $m_{\mathrm{it}}$ made by each resident based on their insurance status, income level, and health status $\theta_{\mathrm{it}}$ . Therefore, by substituting the observed income $y_{\mathrm{it}}$ and medical expenditure $m_{\mathrm{it}}$ from the CFPS into Equation 6 and estimating the unknown parameters, we can derive the estimation of residents’ latent health status. The regional insurance premium $p_{\mathrm{it}}$ for each resident is sourced from official policy documents, and the national average premiums for URRBMI in 2020 and 2022 were 250 yuan and 380 yuan, respectively. The remaining unknowns in Equation 6 are ${\hat{f}}_{\mathrm{jkt}}(a_{\mathrm{it}}|m_{\mathrm{it}})$ , $\frac{\partial {\hat{f}}_{\mathrm{jkt}}(a_{\mathrm{it}}|m_{\mathrm{it}})}{\partial m_{\mathrm{it}}}$ , ${\hat{\gamma }}_{1}$ and ${\hat{\gamma }}_{2}$ , which will be estimated in Section 2.4. Our estimation yields a more accurate and objective assessment of health compared to self-assessed data. Accordingly, we can identify adverse selection by comparing the distribution of the latent health status between insured and uninsured residents, and propose the first hypothesis:

*H_1_:* The latent health status’s cumulative distribution function of the insured is first-order stochastic dominant over the uninsured; that is, the average health level of enrollees is worse than that of the uninsured, meaning that there is adverse selection in the URRBMI enrollment.

According to the price elasticity of demand theory, when the price of goods declines, it will generate two distinct effects. Firstly, the substitution effect occurs because the relative price of the goods decreases, prompting consumers to substitute it for other goods in their purchasing decisions. This effect reflects market distortions caused by price changes, potentially leading to moral hazard and deadweight loss. Secondly, the income effect arises from changes in consumer’s real income due to the price change. A price decrease effectively raises the purchasing power of money (i.e., “hidden income”), which does not inherently distort market behavior but instead releases suppressed demand. In the healthcare market, medical insurance reduces the price of healthcare services faced by the insured, thereby triggering both effects. Notably, the income effect manifests as increased healthcare utilization, representing the normal release of medical demand and an improvement in social welfare. Conversely, the substitution effect drives overutilization of healthcare services due to insurance coverage, which can be quantified as moral hazard (15).

Following the methodology of Bajari et al. (38), Wang and Zhu (40) and Nyman et al. (42), our study also constructs a counterfactual scenario where a resident transitions from insured to uninsured status to quantify the moral hazard in URRBMI. Specifically, to quantify the substitution effect resulting from insurance enrollment, we employ the Slutsky decomposition theory to construct the counterfactual scenario. We maintain the utility function for resident *i* as specified in Equation 2, under the assumption that insurance enrollment affects only the resident’s budget constraint. According to the Slutsky decomposition, the change in the optimal consumption decision when resident *i* transitions from insured to uninsured status is decomposed into the following steps:

(1) Initial Scenario: Assume the resident *i* is enrolled in the URRBMI, with the budget constraint given by Equation 8.

$$c_{i}+(1-a_{i})m_{i}\leq y_{i}-p_{i}$$ (8)

Where the resident *i* faces a relative price of healthcare services to composite goods given by $\frac{P_{m}}{P_{C}}=1-a_{i}$ and a disposable income of $y_{i}-p_{i}$ . Under this constraint, solving the utility maximization problem yields the optimal consumption bundle $\left(c_{1},m_{1}\right)$ .

(2) Decomposition Process 1: Measuring the change in the optimal consumption bundle induced exclusively by the variation in the relative price of goods, while maintaining purchasing power constant. This step corresponds to the measurement of the substitution effect.

First, when resident *i* transitions from an insured to an uninsured status, the relative price of goods increases from $1-a_{i}$ to 1. Second, to hold the resident’s purchasing power unchanged, we compute the compensatory income variation necessary to sustain the initial consumption bundle $\left(c_{1},m_{1}\right)$ , as expressed in Equation 9.


$$\mathrm{\Delta y}=[(\frac{P_{m}}{P_{C}})\prime -(\frac{P_{m}}{P_{C}})]*m_{1}=[1-(1-a_{1})]*m_{1}=a_{1}m_{1}$$ (9)

Under the new budget constraint specified in Equation 10, the optimal consumption bundle obtained by solving the utility maximization problem is $\left(c_{2},m_{2}\right)$ . The magnitude of the substitution effect is given by $\Delta m^{s}=m_{2}-m_{1}$ .


$$c_{i}+m_{i}\leq y_{i}-p_{i}+a_{1}m_{1}$$ (10)

(3) Decomposition Process 2: Measure the change in the demand for healthcare services resulting from the alteration in purchasing power induced by the price change, which constitutes the income effect.

In contrast to Decomposition Process 1, the relative price of goods faced by resident *i* remains increased from $1-a_{i}$ to 1, but the assumption of constant purchasing power is relaxed. The resident’s income maintained at the initial level $y_{i}-p_{i}$ . Under this new budget constraint specified in Equation 11, solving the utility maximization problem yields the optimal consumption bundle $\left(c_{3},m_{3}\right)$ . The magnitude of the income effect is quantified as $\Delta m^{n}=m_{3}-m_{2}$ .


$$c_{i}+m_{i}\leq y_{i}-p_{i}$$ (11)

(4) Final Scenario: Assume the resident *i* is uninsured, facing a relative price of healthcare services to composite goods of 1, and has an income of $y_{i}$ . The budget constraint given by Equation 12.

$$c_{i}+m_{i}\leq y_{i}$$ (12)

Compared to Decomposition Process 2, the final scenario incorporates the direct income increase due to the absence of insurance premium payments, which also contributes to the income effect. Under the budget constraint in Equation 12, utility maximization yields the optimal consumption bundle $\left(c_{4},m_{4}\right)$ . The total income effect is quantified as $\Delta m^{n}=m_{4}-m_{2}$ .

Overall, $\mathrm{\Delta m}=m_{1}-m_{4}\;$ captures the total difference in medical expenditure for the same resident under insured versus uninsured status. This gap can be decomposed into two components: a substitution effect, driven by the change in relative prices, and an income effect, reflecting the combined impact of the price change on purchasing power and the income variation due to premium payment status. The substitution effect, measured by $\Delta m^{s}=m_{2}-m_{1}$ , constitutes the primary focus of our study.

Based on the above analysis, we propose the following approach for estimating moral hazard. First, insured residents from the CFPS are selected as our research subjects, with their observed medical expenditure denoted as $m_{1}$ . By altering their budget constraint to Equation 10 and resolving the utility maximization problem, we construct a counterfactual scenario and obtain the optimal medical expenditure under this counterfactual scenario, denoted as $m_{2}$ . A comparison between $m_{1}$ and $m_{2}$ provides an estimate of the substitution effect, which is interpreted as the measure of moral hazard. It is noteworthy that, as resident’s medical consumption decisions are influenced by both self-selection and the physician’s diagnosis, the moral hazard estimated in this study is derived from both demand-side and supply-side. Accordingly, we propose Hypothesis 2:

*H_2_:* Some residents’ $m_{2}$ is significantly less than their $m_{1}$ , indicating that these residents use less healthcare services and increase the consumption of other goods under the counterfactual scenario; in other words, these residents have a medical over-consumption in the actual scenario and have moral hazard problems.

### 2.4 Estimation

#### 2.4.1 Estimation for conditional probability density

We use the 2020 and 2022 data from CFPS to estimate the conditional probability density ${\hat{f}}_{\mathrm{jkt}}(a_{\mathrm{it}}|m_{\mathrm{it}})$ and the partial derivative $\frac{\partial {\hat{f}}_{\mathrm{jkt}}(a_{\mathrm{it}}|m_{\mathrm{it}})}{\partial m_{\mathrm{it}}}$ in Equation 6 for the first step. Firstly, the ${\hat{f}}_{\mathrm{jkt}}(a_{\mathrm{it}}|m_{\mathrm{it}})$ can be calculated by dividing the estimated by the probability density of medical expenditure, as illustrated in Equation 13:


$${\hat{f}}_{\mathrm{jkt}}(a_{\mathrm{it}}|m_{\mathrm{it}})=\frac{{\hat{f}}_{\mathrm{jkt}}(a_{\mathrm{it}},m_{\mathrm{it}})}{{\hat{f}}_{\mathrm{jkt}}(m_{\mathrm{it}})}$$ (13)

There are two methods to estimate population distribution by the sample data: parametric and nonparametric methods. The former requires assumptions about the specific form of population distribution, such as normal distribution, Poisson distribution, or binomial distribution. And the unknown parameters in the probability density function are then obtained by calculating the sample’s numerical characteristics. Therefore, the accuracy of parameter estimation depends on the rational specification of the distribution function form. To avoid biases caused by functional form misspecification, we employ the nonparametric method of kernel density estimation (KDE) for estimation, which is first proposed by Rosenblatt (43). First, we employ the Epanechnikov kernel, which theoretically minimizes the integrated mean square error (IMSE) as the kernel function for our estimation. Second, given that the medical expenditure data often exhibits a significantly right-skewed distribution in reality, where a small minority bears the majority of medical costs, the modified Silverman’s rule is employed as the basis for selecting the optimal bandwidth (44). The conditional distribution is calculated for a grid that breaks reimbursement percentages into 100 categories. Both more and fewer grids were used, with very little change in the estimates. Finally, we can derive the conditional distributions ${\hat{f}}_{\mathrm{jkt}}(a_{\mathrm{it}}|m_{\mathrm{it}})$ .

However, the results of kernel density estimation are closely related to the choice of the optimal bandwidth (45). If the selected bandwidth is too large, the estimated result becomes overly smooth and may obscure distributional details (such as bimodality). Conversely, if the bandwidth is too small, the estimated curve becomes rugged and more sensitive to noise. To examine the sensitivity of our KDE results, the cross-validation method was further employed to the optimal bandwidth calculation. Taking the estimation of joint probability density ${\hat{f}}_{\mathrm{jkt}}(a_{\mathrm{it}},m_{\mathrm{it}})$ in Equation 13 as an example, Table 3 reports the optimal bandwidth calculated using the modified Silverman rule and the cross-validation method, respectively. It can be observed that for different years, types of medical services, and hospital tiers, the optimal bandwidth values derived from the two criteria show no significant differences, demonstrating the robustness of our estimation results.

**Table 3:** The sensitivity analysis of kernel density estimation.

Year	Method	Outpatient in primary hospital		Outpatient in secondary hospital		Outpatient in tertiary hospital		Inpatient in primary hospital		Inpatient in secondary hospital		Inpatient in tertiary hospital	
		$m_{\mathrm{it}}$	$a_{\mathrm{it}}$	$m_{\mathrm{it}}$	$a_{\mathrm{it}}$	$m_{\mathrm{it}}$	$a_{\mathrm{it}}$	$m_{\mathrm{it}}$	$a_{\mathrm{it}}$	$m_{\mathrm{it}}$	$a_{\mathrm{it}}$	$m_{\mathrm{it}}$	$a_{\mathrm{it}}$
2020	Silverman rule	6.132	0.031	10.107	0.031	12.529	0.026	256.846	0.014	370.608	0.016	408.183	0.022
	Cross-validation	5.279	0.034	12.957	0.035	15.043	0.033	221.051	0.015	352.257	0.020	387.731	0.026
2022	Silverman rule	9.723	0.035	9.492	0.033	12.611	0.029	250.540	0.026	318.907	0.018	379.709	0.021
	Cross-validation	7.646	0.042	7.546	0.041	10.529	0.037	227.346	0.022	299.963	0.021	359.047	0.023

The optimal bandwidths for the joint probability density ${\hat{f}}_{\mathrm{jkt}}(a_{\mathrm{it}},m_{\mathrm{it}})$ are reported in the table, with all values derived from computations performed in R (version 2022).

**Figure 1:**
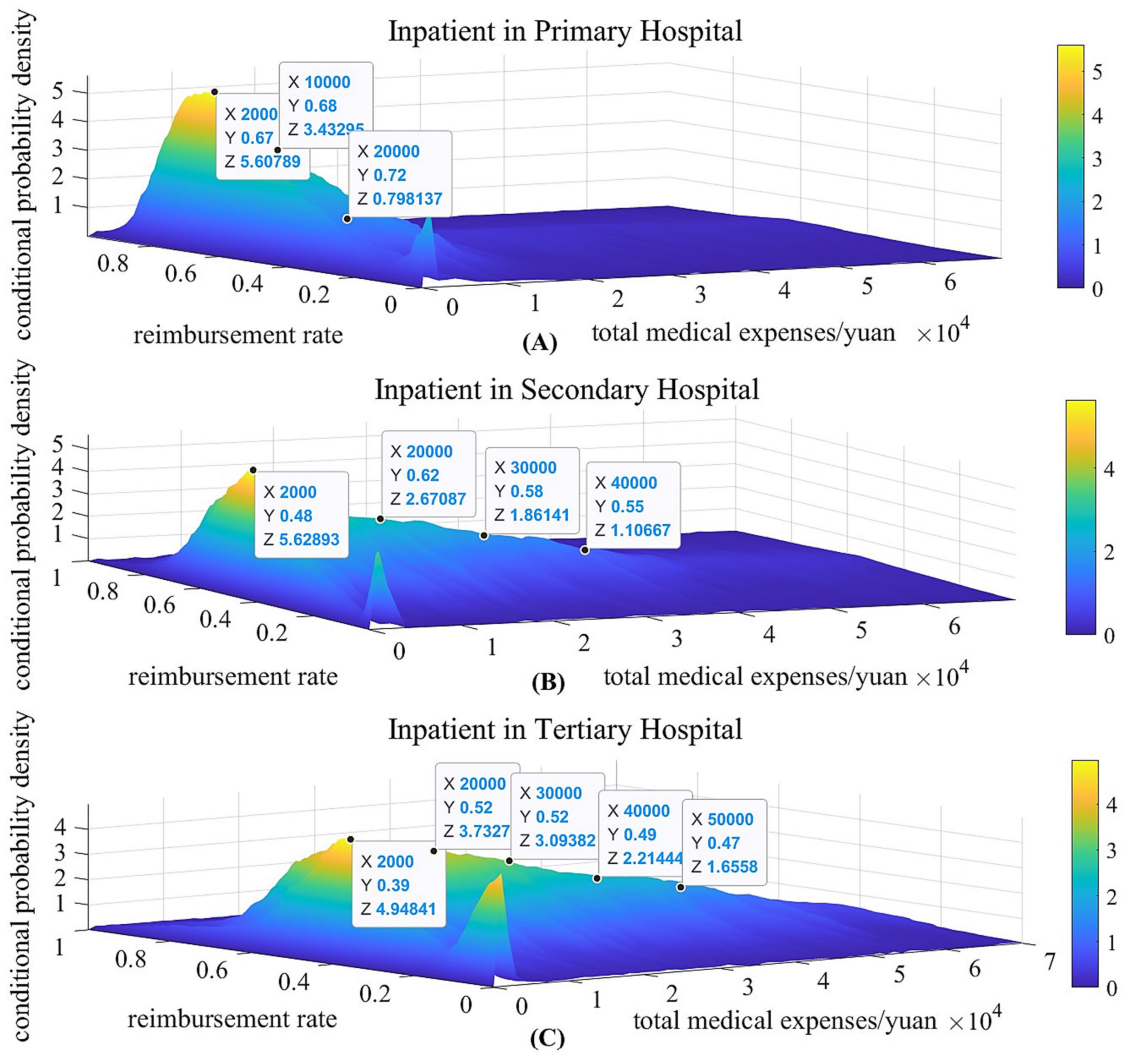
The estimation of the conditional probability density distribution of inpatient in 2022. The x-axis of subfigure **(A, B)** and **(C)** represents the total medical expenses of residents in primary, secondary and tertiary hospital, respectively; The y-axis of subfigure **(A, B)** and **(C)** represents the actual reimbursement rate in primary, secondary and tertiary hospital, which is the ratio of actual insurance reimbursement amount to total medical expenses; The z-axis shows the conditional probability density value.

Taking the hospitalization reimbursement in 2022 as an example, Figures 1A–C, respectively present the estimated probability density distributions of the actual reimbursement rate for inpatient care in primary, secondary, and tertiary hospitals during 2022. The figures reveal that when the resident’s inpatient medical expense is low, the probability of the actual reimbursement rate being zero is consistently highest across all hospital tiers. As medical expenses increases, the actual reimbursement rate exhibits an upward trend, progressively stabilizing. This phenomenon is ​primarily attributable to the deductible threshold provisions in the inpatient reimbursement policy, as discussed in the Section 2.1. However, there are notable differences between the three figures: Firstly​, the probability of incurring high medical expenditure is highest in tertiary hospitals and decreases progressively with lower hospital tiers. Secondly, the actual inpatient reimbursement rate across all expenditure levels demonstrates a declining trend as the hospital tier increases. Specifically, the actual reimbursement rate for primary, secondary, and tertiary hospitals concentrates at approximately 70, 60, and 50%, respectively.

Based on the estimation of the conditional probability density, the partial derivative of conditional probability density with respect to total medical expenditure $\frac{\partial {\hat{f}}_{\mathrm{jkt}}(a_{\mathrm{it}}|m_{\mathrm{it}})}{\partial m_{\mathrm{it}}}$ is then calculated according to the definition of partial derivative in Equation 14.


$$\frac{\partial {\hat{f}}_{\mathrm{jkt}}(a_{\mathrm{it}}|m_{\mathrm{it}})}{\partial m_{\mathrm{it}}}=\lim_{\mathrm{\Delta m}\to 0}\frac{{\hat{f}}_{\mathrm{jkt}}(a_{\mathrm{it}}|m_{\mathrm{it}}+\mathrm{\Delta m})-{\hat{f}}_{\mathrm{jkt}}(a_{\mathrm{it}}|m_{\mathrm{it}})}{\mathrm{\Delta m}}$$ (14)

#### 2.4.2 Estimation for risk aversion coefficients

Subsequently, we substitute the estimates of ${\hat{f}}_{\mathrm{jkt}}(a_{\mathrm{it}}|m_{\mathrm{it}})$ and $\frac{\partial {\hat{f}}_{\mathrm{jkt}}(a_{\mathrm{it}}|m_{\mathrm{it}})}{\partial m_{\mathrm{it}}}$ into Equation 6 to estimate the risk aversion coefficient ${\hat{\gamma }}_{1}$ and ${\hat{\gamma }}_{2}$ for the second step. According to the assumptions outlined in Section 2.3.1, we first select variables related to the risk aversion coefficients to group the population. Based on a review of relevant literatures, we choice gender and individual’s educational level as the grouping variables (41, 46, 47). Specifically, we classify residents with high school education or above as a high-education-level group, while classifying the remainder as a low-education-level group. The gender grouping variable is then incorporated, ultimately resulting in *n* ( n=4 ) subgroups.

In this section, we will implement the moment estimation method to obtain the risk-aversion coefficient estimators ${\hat{\gamma }}_{1n}$ and ${\hat{\gamma }}_{2n}$ . The core idea of this method is to use sample moments to substitute the population moments and then obtain parameter estimates; the difference with the traditional method is that we do not make assumptions about the specific form of the distribution of health status but use the assumption that the distribution of each subgroup is constant between the 2 years, that is, the random variables ${\hat{\theta }}_{20n}$ and ${\hat{\theta }}_{22n}$ have the same distribution. The assumption adopted in our study is consistent with the approach employed by Bajari et al. (38), Zhong et al. (39), Wang and Zhu (40), thus validating its appropriateness. Table 4 also presents the numerical characteristics of the self-assessed health data from the CFPS for waves 2020 and 2022. As illustrated in the table, the first to four central moments of the self-assessed health variable-namely the mean, variance, skewness, and kurtosis-remain relatively stable between the 2 years in each subgroup. And the results of K-S test in Table 4 also indicate that, at the 10% significance level, the null hypothesis that the self-assessed health data across different subgroups share the same distribution between the 2 years cannot be rejected, further validating the hypotheses.

**Table 4:** Descriptive statistics of self-assessed health data in CFPS.

Subgroup	Male & low education level		Male & high education level		Female & low education level		Female & high education level	
Year	2020	2022	2020	2022	2020	2022	2020	2022
Min	1	1	1	1	1	1	1	1
Max	5	5	5	5	5	5	5	5
Mean	2.810	2.761	2.572	2.595	3.057	3.028	2.688	2.722
Variance	1.520	1.542	1.073	1.061	1.596	1.581	0.960	0.951
Skewness	0.152	0.204	0.203	0.223	0.006	0.067	0.178	0.251
Kurtosis	2.277	2.290	2.947	3.105	2.175	2.227	3.301	3.474
*N*	5,978	5,521	2,526	2,271	6,266	5,934	2,257	2,069
K-S Statistic	0.017 (0.894)		0.018 (0.283)		0.028 (0.157)		0.014 (0.986)	

1–5 represent five levels of self-assessed health status, with the level of health decreasing as the level increases; The self-assessed health status value of 1 represents “very healthy,” 2 represents “quite healthy,” 3 represents “relatively healthy,” 4 represents “fair,” and 5 represents “unhealthy.”; The null hypothesis of K-S test is the self-assessed health data in 2020 and 2022 have the same distribution, and the alternative hypothesis is that the data in 2020 and 2022 do not have the same distribution, the p value in parentheses.

Equation 15 is the formula for the mean of the health variables $\theta_{\mathrm{it}}$ of group *n* in year *t*, where $N_{\mathrm{nt}}$ is the resident’s number ( n=1,2,3,4 ; *t* = 2020 and 2020). The first moment condition Equation 16 is formulated based on the difference in the mean of the health variables between 2020 and 2022, where $\omega =(m_{\mathrm{it}},y_{\mathrm{it}},p_{\mathrm{it}},{\hat{f}}_{\mathrm{jkt}})$ .


$$\mu_{\theta_{\mathrm{nt}}}(\gamma_{1n},\gamma_{2n})=\sum_{i=1}^{N_{\mathrm{nt}}}\frac{1}{N_{\mathrm{nt}}}\theta_{\mathrm{it}}(\gamma_{1n},\gamma_{2n})$$ (15)

$$h_{1}(\omega ,\gamma_{1n},\gamma_{2n})=\mu_{{\hat{\theta }}_{22n}}(\gamma_{1n},\gamma_{2n})-\mu_{{\hat{\theta }}_{20n}}(\gamma_{1n},\gamma_{2n})$$ (16)

Similarly, three other moment conditions can be constructed using the variance, skewness, and kurtosis, respectively. The moment conditions $h_{2}$ , $h_{3}$ , and $h_{4}$ are expressed in terms of the differences in variance, kurtosis, and skewness of the health variables between 2022 and 2020.

Given that the health distributions of different subgroups remain consistent over the two period, it follows that $E[h_{l}(\omega ,\gamma )]=0$ ( l=1,2,3,4 ). The moment estimator ${\hat{\gamma }}_{1n}$ and ${\hat{\gamma }}_{2n}$ is defined as the value that minimizes the sum of all four squared sample moment conditions, which can be written as the objective function Equation 17. Referring to Bajari et al. (38), the value intervals of the risk aversion coefficients in this study were set as: $\gamma_{1n}\in [1,9]$ and $\gamma_{2n}\in [1,9]$ . We divide equally grids on the plane consisting of their possible values, and then substitute each combination of grid values for $\;\gamma_{1n}$ and $\gamma_{2n}$ into the sample moments. The optimal estimate is the one which makes the sum closest to zero.


$$[{\hat{\gamma }}_{1n},{\hat{\gamma }}_{2n}]=\text{argmin}\sum_{l=1}^{4}h_{l}^{2}$$ (17)

Table 5 illustrates the estimation of risk aversion coefficients of subgroups, and the standard errors in parentheses are obtained by bootstrap iterations. Bootstrapping is a self-service resampling method that creates a “bootstrap sample” with the same size as the original sample by sampling with the replacement. Then this process is repeated several times to obtain a set of bootstrap samples, which are used for statistical inferences. This method is used independent of the population distribution and does not require assumptions about it, which can effectively avoid the problem of invalid estimates due to incorrect distribution assumptions. We conduct 100 bootstrap iterations and perform the grid search under three different grid sizes. The mean value of the estimates from 100 iterations is taken as the final coefficient estimates under different grid sizes.

**Table 5:** Estimation for risk aversion coefficients.

Grids	Male & low education level			Male & high education level			Female & low education level			Female & high education level		
	20*20	40*40	50*50	20*20	40*40	50*50	20*20	40*40	50*50	20*20	40*40	50*50
$\gamma_{1}$	4.079 (1.005)	4.256 (0.945)	3.867 (0.804)	5.421 (2.053)	5.069 (1.976)	5.357 (2.028)	5.251 (1.575)	4.969 (1.342)	5.354 (1.045)	5.545 (1.765)	5.769 (1.845)	5.835 (2.118)
$\gamma_{2}$	6.059 (1.073)	5.821 (1.379)	6.001 (1.505)	7.048 (2.305)	7.274 (1.907)	7.141 (2.341)	6.747 (1.045)	6.923 (1.170)	6.802 (1.027)	7.828 (2.115)	8.020 (1.789)	7.958 (1.959)
*N*	11,499			4,797			12,200			4,326		

Standard errors in parentheses.

The estimates derived from varying grid sizes show no significant differences within subgroups; therefore, the average of the three grid-size estimates is used as the final measure. Specifically, the risk aversion coefficients for composite goods and medical expenditures are 4.067 and 5.960 for the male low-education group, and 5.282 and 7.154 for the male high-education group. Corresponding values for the female low-education group are 5.191 and 6.823, and for the female high-education group, 5.716 and 7.935. The results indicate, first, that risk aversion coefficients for medical expenditures are systematically higher than those for composite goods. A higher medical utilization risk aversion coefficient is consistent with the notion that individuals are more risk averse with respect to their health, as it often cannot be regained once lost. Second, significant variations exist across subgroups. Females show higher risk aversion than males for both categories of consumption, a result aligned with the findings of Jianakoplos and Bernasek (47). Additionally, higher education is associated with stronger risk aversion across consumption types, which corroborates intuitive expectations.

## 3 Results

### 3.1 Test for adverse selection in URRBMI

Substituting the estimates in Section 2.4 into Equation 6, we can finally calculate the latent health status of each resident. Provides descriptive statistics for the latent health status of the overall sample, the insured and the uninsured in 2020 and 2022, respectively. The columns (1) and (4) of Table 6 reveal that the mean of residents’ health status were 0.232 in 2020 and 0.252 in 2022, with both years exhibiting identical minimum and maximum values of 0 and 1, suggesting no statistically significant differences in health distribution between the two periods. Table 6 also shows that the mean of health status of the insured ${\hat{\theta }}_{\text{urrmi}}$ is greater than that of the uninsured ${\hat{\theta }}_{\mathrm{nmi}}$ in both 2020 and 2022. In other words, the insured exhibit significantly lower average health levels compared to the uninsured, confirming the presence of adverse selection in both 2020 and 2022. A longitudinal comparison across years further reveals that in 2020, the mean of ${\hat{\theta }}_{\text{urrmi}}$ and ${\hat{\theta }}_{\mathrm{nmi}}$ were 0.239 and 0.192, respectively-only a 0.047 difference. By 2022, the mean of health status shifted to 0.263 for insured and 0.145 for the uninsured, with the gap statistically expanding to 0.118. This phenomenon indicates that relatively healthier residents increasingly opted out of insurance coverage in 2022 compared to 2020, suggesting exacerbated adverse selection issues during the 2022 enrollment.

**Table 6:** Descriptive statistics of latent health status’s estimations.

	${\hat{\theta }}_{20}$	${\hat{\theta }}_{20\text{urrmi}}$	${\hat{\theta }}_{20\mathrm{nmi}}$	${\hat{\theta }}_{22}$	${\hat{\theta }}_{22\text{urrmi}}$	${\hat{\theta }}_{22\mathrm{nmi}}$
Min	0	0	0	0	0	0
Max	1	1	1	1	1	1
Mean	0.232	0.239	0.192	0.252	0.263	0.145
Std. Dev	0.404	0.407	0.376	0.415	0.421	0.335
*N*	17,027	14,756	2,271	15,795	14,241	1,554

${\hat{\theta }}_{20}$ , ${\hat{\theta }}_{20\text{urrmi}}$ and ${\hat{\theta }}_{20\mathrm{nmi}}\;$ denote the latent health status of the overall, insured, and uninsured samples in 2020, respectively; ${\hat{\theta }}_{22}$ , ${\hat{\theta }}_{22\text{urrmi}}$ and ${\hat{\theta }}_{22\mathrm{nmi}}$ denote the latent health status of the overall, insured, and uninsured samples in 2022, respectively.

**Figure 2:**
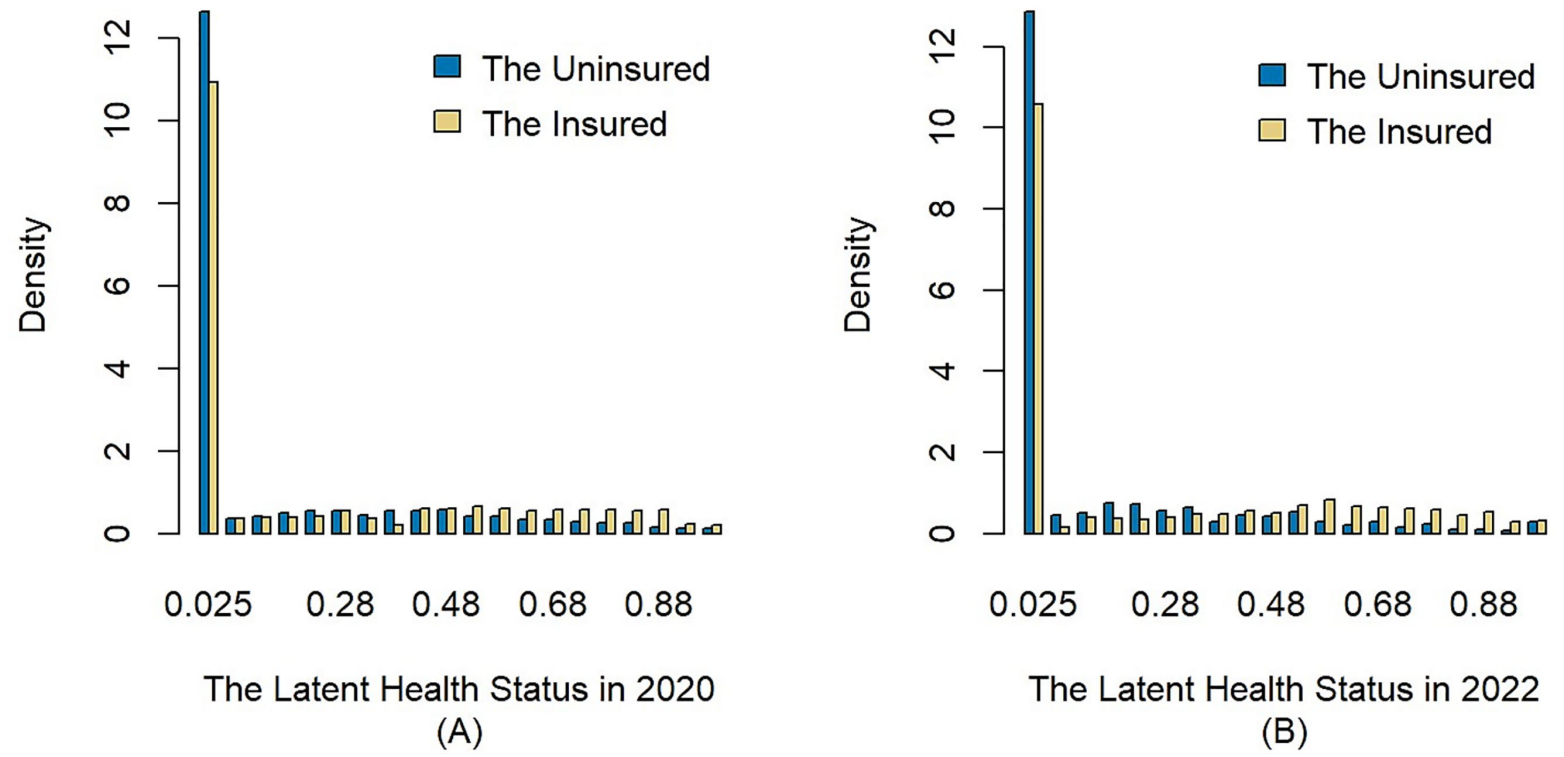
Distribution curves of latent health status. The x-axis of subfigure **(A)** and **(B)** represents the latent health status of samples in 2020 and 2022, respectively; The y-axis shows the value of probability density; The blue bars represent the samples without medical insurance, and the yellow bars represent the samples enrolled in the URRBMI.

Figure 2 further plots the distribution histograms of latent health status for the insured and uninsured in 2020 and 2022. As illustrated, both in 2020 and 2022, the group with a latent health status value of 0 accounts for the highest proportion, indicating that most of the samples are in good health with no medical visits within the year, which is consistent with the real-world situation. Secondly, the value of latent health status is denser near 1 and less dense near 0 for the insured group compared to the uninsured group. This indicates that more residents with poor health are pooled in the insured group. Furthermore, Table 7 illustrates the results of K-S test. The results reject the null hypothesis and support that the health distribution function of the insured lies below that of the uninsured at the 1% significance level. Combining the Table 6, Figure 2 with the K-S test results, it can be concluded that the latent health status of the insured has first-order stochastic dominance over the uninsured. Thus, Hypothesis 1 is accepted; that is, the average health level of the insured is lower than the uninsured, which means there is adverse selection in the URRBMI.

**Table 7:** Two-sample Kolmogorov–Smirnov test.

Year	K-S statistic	*p* value	Null hypothesis	Alternative hypothesis	Outcome
2020	0.123	0.000	$\theta_{\text{urrmi}}$ and $\theta_{\mathrm{nmi}}\;$ have the same distribution	The CDF of $\theta_{\text{urrmi}}$ lies below that of $\theta_{\mathrm{nmi}}$	Reject the null hypothesis
2022	0.188	0.000			Reject the null hypothesis

### 3.2 Test for moral hazard in URRBMI

We employ the counterfactual scenario established in Section 2.3.3 to assess the presence and magnitude of moral hazard. If the optimal medical expenditure derived under the counterfactual scenario is lower than the resident’s actual medical expenditure, confirming the presence of moral hazard. Furthermore, the difference between actual medical expenditure and counterfactual expenditure can serve as an estimate of moral hazard.

Table 8 provides descriptive statistics on the actual medical expenditures that exceed the counterfactual expenditures for the insured samples in 2020 and 2022. Among the 14,756 samples enrolled in 2020, 46.63% of patients had actual medical expenditures exceeding their counterfactual medical expenditures. This proportion decreased to 44.76% in 2022, suggesting that this segment of enrollees may had an over-consumption of medical utilization. On average, this behavior increased resident’s medical expenditure by 204.57 yuan, 13.8% of the actual medical expenditure in 2020, and by 187.631 yuan, 10.6% of the actual medical expenditure in 2022. The findings show no significant differences compared to the results of relevant empirical research and do not exhibit extreme estimates, thereby confirming the plausibility of our findings (14, 16, 22, 26, 40). Based on the results, our study identifies the presence of moral hazard in URRBMI; thus, Hypothesis 2 is accepted. Additionally, a comparison of the moral hazard between 2020 and 2022 reveals a significant decline in its magnitude by 2022. Specifically, the proportion of medical over-consumption to the actual medical expenditures decreased by approximately 3.2. This phenomenon may be associated with the implementation of China’s Diagnosis-Related Groups (DRG) and Diagnosis-Intervention Packet (DIP) payment reforms since 2020. Section 3.3 will further analyze the correlation between these reforms and the observed decline in moral hazard, exploring the underlying mechanisms.

**Table 8:** Descriptive statistics of medical over-consumption.

		Actual medical expenditure	Counterfactual medical expenditure	Medical over-consumption	Medical over-consumption share
2020	Min	2	1.714	0.286	0.000
	Max	55,000	52,922.050	5,275.519	0.226
	Mean	1,693.258	1,488.688	204.570	0.138
	Std. Dev	2,837.256	2,555.788	316.603	0.024
	*N*	6,880	6,880	6,880	6,880
2022	Min	1	0.887	0.112	0.000
	Max	80,000	75,690.030	5,795.035	0.201
	Mean	2,100.173	1,912.542	187.631	0.106
	Std. Dev	4,183.504	3,872.021	353.621	0.024
	*N*	6,374	6,374	6,374	6,374

Medical over-consumption is the proportion of actual medical expenditure higher than counterfactual medical expenditure, serving as a measure of moral hazard; Medical over-consumption share is the amount of medical over-consumption as a proportion of the actual medical expenditure.

**Figure 3:**
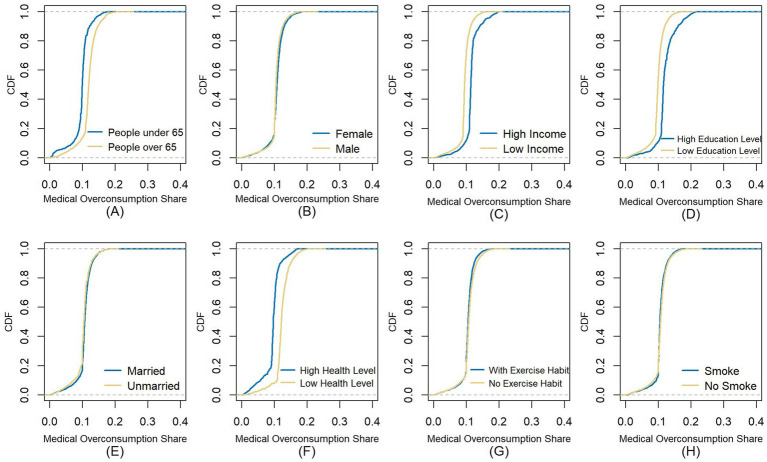
The x-axis represents the medical over-consumption share, and the y-axis shows the value of the cumulative distribution function; We divide samples into high-income and low-income groups according to whether income levels exceed the overall average; We classify individuals with high school education or above as a high-education-level group, while classifying the remainder as a low-education-level group; Using the resident’s actual health status measured in Section 3.1, samples are divided into two groups based on the mean of overall health levels; Residents who reported never engaging in physical exercise in the past 12 months are categorized as a no-exercise-habit group, with the remainder classified as an exercise-habit group; We employ “whether smoked in the past month” as the measurement criterion for resident’s smoking habit.

Prior studies indicate that moral hazard is associated with individual characteristics such as gender, income, health status, education, and marital status. We therefore examine heterogeneity in moral hazard by grouping individuals based on these socioeconomic and demographic factors, additionally incorporating the variables of individuals’ health risk awareness-proxied by exercise and smoking habits. Figure 3 plots the empirical cumulative distribution function of medical over-consumption share in each group. Subfigures (A)–(E) show similar distributions by gender and marital status, but significant differences by age, income, education, and health status. For example, subfigure (A) reveals first-order stochastic dominance in the group with people over 65, indicating more severe moral hazard relative to younger individuals. Similarly, the moral hazard problem is more severe among groups with higher incomes, higher education levels, or poorer health status. Additionally, subfigures (F) and (G) show no significant difference by health habits. This can be primarily attributed to the fact that our estimation reflects the ex-post moral hazard, which may uncorrelated with these ex-ante health risk variables.

### 3.3 Further discussion of moral hazard

Moral hazard in medical insurance, particularly its extent and manifestations on the supply side, is closely linked to the payment method of medical insurance (48). In 2021, the National Healthcare Security Administration of China formulated the “Three-Year Action Plan for DRG/DIP Payment Reform,” requiring all coordinated regions to implement the reform by 2024 (49). Since then, multiple provinces introduced their local DRG/DIP payment reform measures in 2022. As the sample selected in our study spans the periods before and after the reform, and the estimation of moral hazard showed significant changes between the 2 years. We further employ the DID regression to analyze the causal relationship between payment reform and the decline in moral hazard, in order to provide targeted recommendations for addressing the moral hazard issues. We identify 21 provinces as the treatment group, where the reform had been implemented in most of their city-level coordinated regions by the end of 2022. The remaining 10 provinces, serve as the control group. The DID regression model is specified as Equation 18:


$$Y_{\mathrm{ipt}}=\alpha_{0}+\alpha_{1}\text{Trea}t_{p}*\mathrm{Pos}t_{t}+X_{\mathrm{ipt}}^{\prime }\theta +Z_{\mathrm{pt}}^{\prime }\vartheta +\lambda_{t}+\zeta_{\mathrm{ipt}}$$ (18)

$Y_{\mathrm{ipt}}$ is the dependent variable, representing the medical over-consumption induced by moral hazard for resident *i* at province *p* in year *t*, which can derive from Section 3.2. In order to investigate the influence mechanism, $Y_{\mathrm{ipt}}$ also include the resident’s outpatient and inpatient expenditure, fund payment, and out-of-pocket payment; $\text{Trea}t_{p}$ is a dummy variable indicating whether province *p* implemented the reform in 2022 (taking 1 if reform implemented, and 0 otherwise); $\mathrm{Pos}t_{t}$ is a time dummy variable, taking the value 0 for 2020 and 1 for 2022; $X_{\mathrm{ipt}}$ and $Z_{\mathrm{pt}}$ are the individual-level and province-level control variables; $\lambda_{t}$ denotes the time-fixed effects and $\zeta_{\mathrm{ipt}}$ is the random error term. The coefficient $\alpha_{1}$ captures the net effect of the reform on the dependent variables.

However, the reform implementation across provinces was not randomly assigned but a result of governmental arrangements based the local medical and economic conditions. These factors may also affect the dependent variables, thereby creating the endogeneity issues. For example, economically developed regions may contain larger populations with higher income or education levels, that may relate to the moral hazard. To address this issue, we apply the Propensity Score Matching (PSM) method to construct a control group with individual characteristics similar to the treatment group, then perform the DID regression using the matched sample.

The procedures of PSM are as follows: First, we estimate the propensity score using a logit model, which represents the conditional probability $p(X_{i})=P(\text{Trea}t_{i}=1|X=X_{i})$ that resident *i* is assigned to the treatment group given the covariates $X_{i}$ . The covariates we select are listed in Table 9, which are the observable variables that influence whether an individual enters the treatment group and may also affect the outcome variable. Finally, we perform the one-to-one nearest neighbor matching using the propensity score as the distance metric.

**Table 9:** Selection and definition of covariates.

Variable type	Variable name	Variable definition
Provincial characteristic	Medical level	Number of medical personnel per 1,000 population
		Number of general practitioners per 10,000 population
		Number of hospital beds per 1,000 population
	Economic level	The natural logarithm of per capita GDP
	Financial pressure on medical insurance fund	The revenue-to-expenditure ratio of UEBMI
		The revenue-to-expenditure ratio of URRBMI
Individual characteristic	Basic individual characteristics	Age, Gender, Income, Educational level, Marital status, Number of children, Insurance type, Health status
	Health habit variables	Smoking habit (Measured by whether the individual smoked in the past month; Yes/No)
		Drinking habit (Measured by whether the individual consumed alcohol in the past 3 months; Yes/No)
		Exercise habit (Measured by whether the individual engaged in physical exercise in the past year; Yes/No)
		Habit of taking a midday nap (Yes/No)
		Habit of staying up late (Defined as going to bed after 11:00 p.m.; Yes/No)

**Figure 4:**
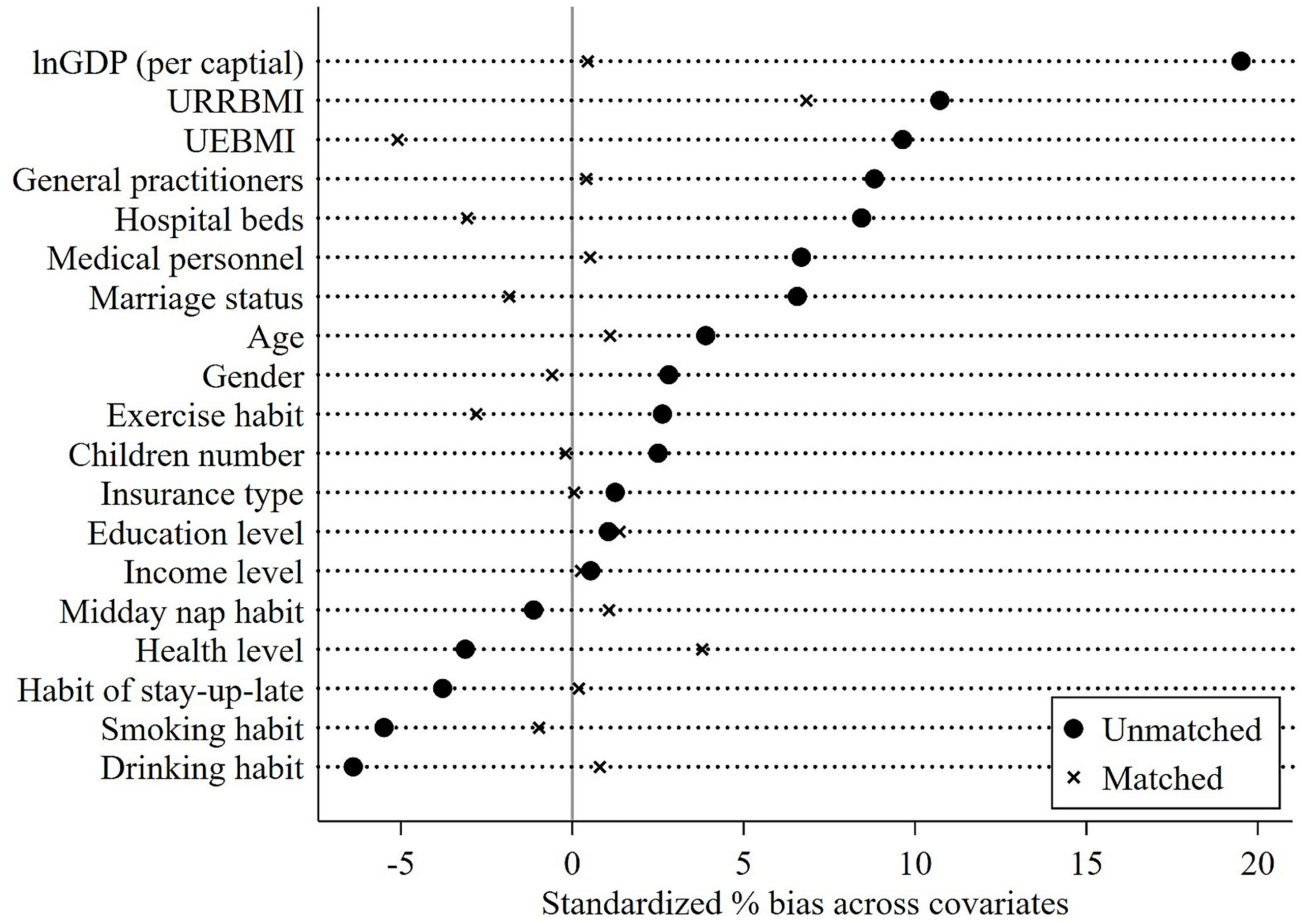
Plot for covariate balance test. The UEBMI and URRBMI in this figure represent the revenue-to-expenditure ratio of Urban Employee Basic Medical Insurance fund and Urban and Rural Resident Basic Medical Insurance fund, respectively.

**Figure 5:**
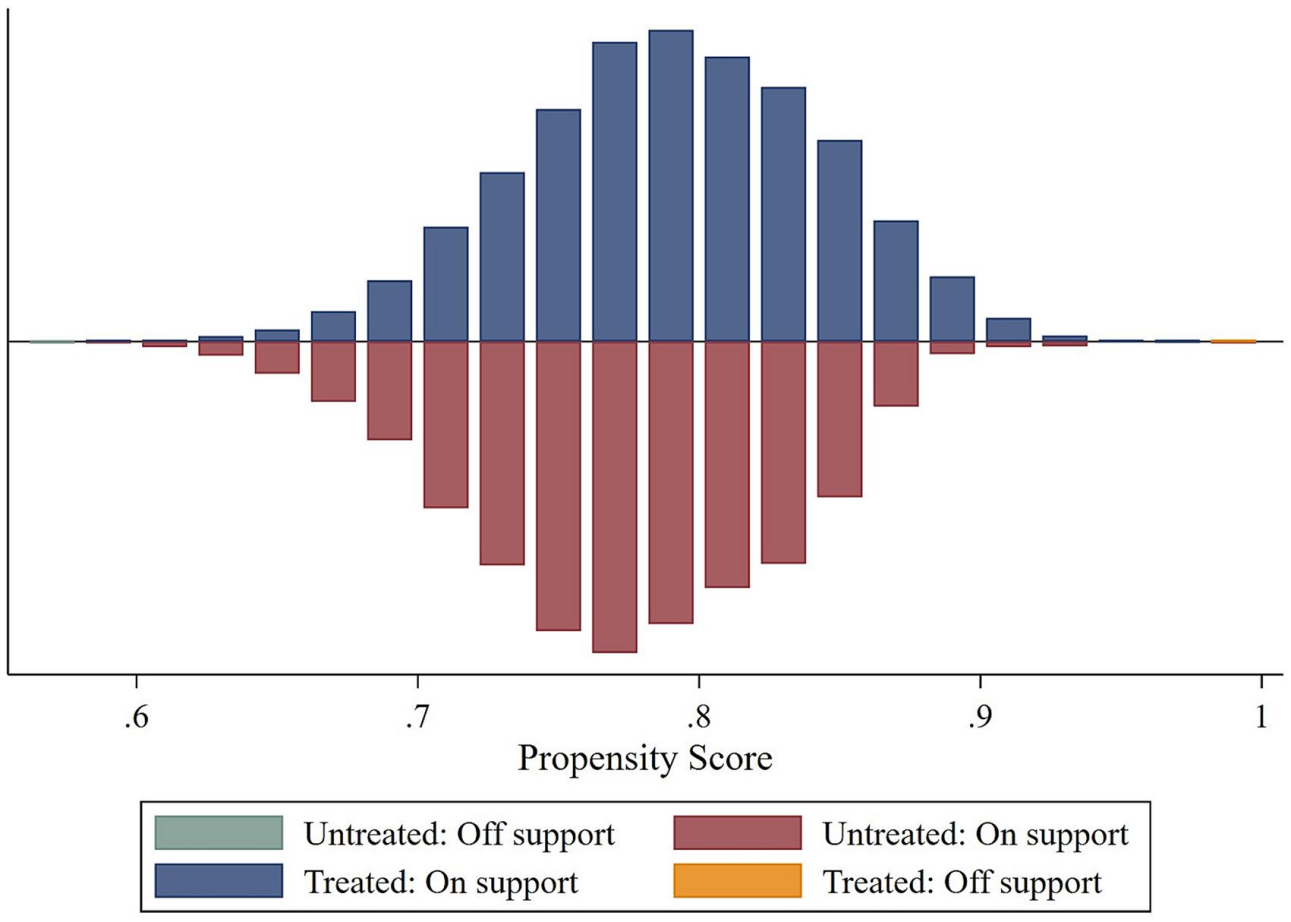
Plot for common support assumption test.

Figure 4 presents the covariate balance test results, showing a substantial reduction in differences between the two groups across most covariates after matching, which indicates satisfactory matching quality. As shown in Figure 5, the common support test confirms that most observations in both the treatment and control groups remain within the common support region, implying that the matching procedure avoided a significant loss of sample size. Using the matched sample constrained to the common support range, we conducted a DID regression. The results are reported in Table 10.

**Table 10:** DID regression results on the impact of medical insurance payment reform.

	(1)	(2)	(3)	(4)	(5)	(6)	(7)	(8)
	Medical over-consumption	Medical over-consumption share	Outpatient expenditure	Outpatient fund payment	Outpatient out-of-pocket	Inpatient expenditure	Inpatient fund payment	Inpatient out-of-pocket
$\text{Trea}t_{p}*\mathrm{Pos}t_{t}$	−0.074* (0.042)	−0.020*** (0.001)	0.036** (0.003)	−0.067 (0.080)	0.042** (0.008)	−0.146* (0.076)	−0.157** (0.066)	−0.031 (0.069)
Control variables	Yes	Yes	Yes	Yes	Yes	Yes	Yes	Yes
Year FE	Yes	Yes	Yes	Yes	Yes	Yes	Yes	Yes
Constant	0.939 (0.586)	0.241*** (0.011)	3.059*** (0.745)	−17.717*** (1.129)	8.216*** (0.819)	−0.497 (1.071)	−0.363 (0.935)	−0.584 (0.979)
*N*	13,241	13,241	13,241	13,241	13,241	13,241	13,241	13,241

The medical over-consumption is log-transformed (natural logarithm) in the regression to mitigate potential heteroscedasticity; And to avoid data loss, we add one to the total medical expenditure, fund payment and the out-of-pocket payment before taking the logarithm in the regression; The resulting estimated coefficients can be interpreted as the percentage change in the dependent variables induced by the reform. Standard errors in parentheses and the “*, **, ***” indicate that the coefficients are significant at 10%, 5%, and 1% significance levels, respectively.

Columns (1) and (2) of Table 10 report the impact of the reform on the amount of medical over-consumption and its share in actual medical expenditure, respectively. The results demonstrate that the 2022 medical insurance payment reform significantly reduced moral hazard-induced overuse, decreasing it by 7.4% and its share by 0.02 percentage points. Further analysis of outpatient and inpatient expenditures shows that the reform reduced inpatient insurance fund payments and total inpatient costs by 14.6 and 15.7%, respectively. In contrast, it raised patients’ out-of-pocket payments and total outpatient costs by 4.2 and 3.6%, while having no statistically significant impact on outpatient insurance fund payments.

To interpret these findings, we briefly explain the mechanisms of DRG/DIP payment system. First, the reform applies exclusively to inpatient care. Taking DRG payment as an example, patients within the same diagnostic group are subject to a fixed payment standard, and any expenses exceeding the standard must be borne by healthcare providers. Under this system, physicians will face the financial risk associated with the excessive prescribing of medications or examinations for the inpatients, which can significantly mitigate the supplier-side moral hazard. Second, to alleviate financial pressures, physicians may shift certain services, such as the pre-admission examinations, treatments for mild cases, and post-discharge management to outpatient departments, leading to spillover effects on outpatient costs. The DID regression confirms the significant role of payment reform in reduced both inpatient insurance expenditures and total costs, demonstrating the reform’s effectiveness. On the other hand, it also indicates that the reform exerts a minor spillover effect on outpatient expenses. Since our measure of moral hazard encompasses both outpatient and inpatient services, the results further reveal that the reform primarily curbs moral hazard by reducing insurance expenditures on inpatient services, effectively standardizing the physician behavior.

## 4 Discussion

In this study, we estimated adverse selection and moral hazard within the URRBMI under a unified analytical framework. First, we constructed and solved a utility maximization model for resident’s medical consumption, and then estimated the unknown variables and parameters using the kernel density estimation, moment estimation and bootstrap methods. This allowed us to derive an estimate of resident’s latent health status. The adverse selection was tested by comparing the health status distributions between insured and uninsured groups. By comparing the distribution of health status between the insured and the uninsured, we obtained evidence of adverse selection both in 2020 and 2022. Additionally, despite enhanced reimbursement benefits, the adverse selection intensified in 2022, largely due to a concurrent substantial increase in individual premium payments.

Subsequently, based on our model and the Slutsky decomposition theory, we constructed a counterfactual scenario to measure the moral hazard in URRBMI. By comparing counterfactual with actual medical expenditures, we identified significant moral hazard in both 2020 and 2022. The excessive medical consumption attributed to moral hazard averaged 204.57 yuan in 2020, accounting for 13.8% of the actual spending. By 2022, this proportion declined to 10.6%. DID regression further confirm that the reduction in moral hazard is associated with the payment system reform of DRG/DIP initiated in 2022. By curbing inpatient insurance expenditures, the reform has standardized physicians’ diagnostic and therapeutic behaviors, thereby effectively mitigating the moral hazard. Finally, the heterogeneity test results suggested that the moral hazard was more severe among enrollees who were older adults, had higher incomes, were in poorer health, or had higher education levels.

Based on our findings, we propose the following recommendations to mitigate excessive medical resources consumption and strengthen China’s medical insurance system. First, although the reimbursement benefits of URRBMI have improved, the substantial increase in individual premiums has considerably dampened enrollment willingness among healthier residents. The government should therefore enhance premium subsidies while continuing to improve benefits. Second, since the URRBMI enrollment relies heavily on mobilization by communities or village committees, these entities can heighten residents’ awareness of health risks alongside their understanding of medical insurance through outreach campaigns and household education sessions, thereby intensifying enrollment mobilization efforts. Although expanding insurance coverage can improve population health, it may also exacerbate excessive medical resources consumption due to moral hazard. Therefore, China should strengthen the promotion of payment reforms, continuously optimize them during implementation, and ultimately establish a comprehensive, efficient, and sustainable payment mechanism.

In this study, we creatively used the model to estimate and compare the latent health status of residents when testing adverse selection, which is more accurate than using subjective data from questionnaires. Furthermore, in estimating the model’s unknown parameters, we diverged from prior researches by employing the semi-parametric estimation approach. This method not only allowed us to flexibly specify the insurance reimbursement schedules but also avoided the bias caused by function mis-setting. Finally, to test moral hazard, the behavioral changes of residents after enrollment were studied by constructing a counterfactual scenario. On the one hand, we assumed that the resident’s health status did not change in the counterfactual scenario. Therefore, there was no adverse selection among the factors that may lead to this excessive medical resources consumption. On the other hand, we employed the Slutsky decomposition to eliminate the influence of the income effect, thereby excluding the impact of the release of normal medical demand due to insurance enrollment on the estimation of moral hazard. The methodological approach adopted in our study yields accurate estimates of both adverse selection and moral hazard, offering a conceptual framework for subsequent researches.

## 5 Limitation

Our study also has some limitations. First, we employed a grouped estimation method in the process of risk aversion coefficient estimation. Although this method can, to some extent, reflect the heterogeneity of risk aversion coefficients across different groups, it still falls short of fully explaining the differences in risk aversion coefficients among individuals. Additionally, the selection of grouping variables for risk aversion coefficient is based solely on relevant literatures, without conducting a more precise selection through correlation analysis of the factors influencing the coefficients. And the grouping method also demands a large sample size. While more refined grouping can better approximate real-world conditions, it simultaneously reduces the number of samples in each subgroup, compromising estimation validity. Due to sample size constraints, we only selected two characteristic variables for grouping. Overall, the rough selection of grouping variables and the small sample size are key limitations of the grouping method and represent an important direction for future research optimization.

Second, when investigating the causal relationship between the DRG/DIP payment reform and the decline in moral hazard, our study divides the sample into treatment and control groups based on whether the province where the sample is located has implemented the payment reform. However, in some provinces, the reform has been rolled out gradually-i.e., piloted in certain cities before being expanded across the province. As a result, classifying treatment and control groups at the city level would be more accurate. The CFPS database does not publicly release the district or county-level data to maximize the protection of respondents’ privacy. The unavailability of this data thus constitutes a significant limitation for this part of the research. When such data becomes available, this represents a potential direction for future research refinement. Additionally, due to the limited availability of variables, we only provide a preliminary exploration of the mechanism through which the payment reform affects moral hazard. However, the impact of payment reform also encompasses many other dimensions, such as its influence on the types and costs of medications and examination prescribed by physicians, as well as potential issues such as “upcoding” and “admission splitting.” These factors may all affect the degree of moral hazard. Research on the correlation between medical insurance payment reform and moral hazard is currently a key topic and warrants further in-depth investigation.

## Data Availability

Publicly available datasets were analyzed in this study. This data can be found at: https://www.isss.pku.edu.cn/cfps/.

## Footnotes

1 For the uninsured: $p_{\mathrm{it}}=0$ , ${\hat{f}}_{\mathrm{jkt}}(a_{\mathrm{it}}|m_{\mathrm{it}})=0$ and $\frac{\partial {\hat{f}}_{\mathrm{jkt}}(a_{\mathrm{it}}|m_{\mathrm{it}})}{\partial m_{\mathrm{it}}}=0$ , $\forall a_{\mathrm{it}}\in [0,1]$ .

## References

[1] General Office of the State Council. Opinions of the state council on integrating the basic medical insurance system for urban and rural residents. (2016). Available online at: https://www.gov.cn/gongbao/content/2016/content_5036268.htm (Accessed January 3, 2016).

[2] Arrow KJ. Uncertainy and the welfare economics of Medicare care. Am Econ Rev. (1963) 53:941–73. doi: 10.2307/1812044

[3] Finkelstein A McGarry K. Multiple dimensions of private information: evidence from the long-term. Am Econ Rev. (2006) 96:938–58. doi: 10.1257/aer.96.4.938, 21253439 PMC3022330

[4] Dave D Kaestner R. Health insurance and ex-ante moral hazard: evidence from Medicare. Int J Health Care Financ Econ. (2009) 9:367–90. doi: 10.1007/s10754-009-9056-4, 19277859

[5] Konetzka RT He D Dong J Nyman JA. Moral hazard and long-term care insurance. Geneva Pap Risk Insur Issues Pract. (2019) 44:231–51. doi: 10.1057/s41288-018-00119-1, 40196840 PMC11974411

[6] Ettner SL. Adverse selection and the purchase of Medigap insurance by the elderly. J Health Econ. (1997) 16:543–62. doi: 10.1016/S0167-6296(97)00011-8, 10175630

[7] Cohen A Siegelman P. Testing for adverse selection in insurance markets. J Risk Insur. (2010) 77:39–84. doi: 10.1111/j.1539-6975.2009.01337.x

[8] Bo H Zhang YH. A study on adverse selection of supplemental commercial medical insurance: evidence based on CHARLS data. Insur Stud. (2015) 9:65–81. doi: 10.13497/j.cnki.is.2015.09.006

[9] Fan QZ Sun QX. Is there adverse selection in China's life insurance market? Empirical evidence from CHARLS data. J Financ Res. (2020) 8:112–29.

[10] Chiappori PA Salanie B. Testing for asymmetric information in insurance markets. J Polit Econ. (2000) 108:56–78. doi: 10.1086/262111

[11] Keane M Stavrunova O. Adverse selection, moral hazard and the demand for medigap insurance. J Econ. (2016) 190:62–78. doi: 10.1016/j.jeconom.2015.08.002

[12] Sengupta R Rooj D. The effect of health insurance on hospitalization: identification of adverse selection, moral hazard and the vulnerable population in the Indian healthcare market. World Dev. (2019) 122:110–29. doi: 10.1016/j.worlddev.2019.05.012

[13] Alessie RJ Angelini V Mierau JO Viluma L. Moral hazard and selection for voluntary deductibles. Health Econ. (2020) 29:1251–69. doi: 10.1002/hec.4134, 32734647 PMC7539990

[14] Xie MM Wang MJ Xiong XJ. Moral hazard or release of medical demand? -medical insurance and growing medical costs. Insur Stud. (2016) 1:102–12. doi: 10.13497/j.cnki.is.2016.01.010

[15] Huang W Ta YQ. Moral hazard in health insurance: evidence from China. J China Econ. (2022) 1:159–190+376-378.

[16] Zhu ML Wang EN. Normal health demand, patient moral hazard or supplier induced demand? The source of rising medical expenditure after the change of basic medical insurance type. Econ Sci. (2021) 2:110–22.

[17] Manning WG Newhouse JP Duan N Keeler EB Leibowitz A. Health insurance and the demand for medical care: evidence from a randomized experiment. Am Econ Rev. (1987) 77:251–77. doi: 10.2307/1804094, 10284091

[18] Aron-Dine A Einav L Finkelstein A. The RAND health insurance experiment, three decades later. J Econ Perspect. (2013) 27:197–222. doi: 10.1257/jep.27.1.197, 24610973 PMC3943162

[19] Chiappori PA Durand F Geoffard PY. Moral hazard and the demand for physician services: first lessons from a French natural experiment. Eur Econ Rev. (1998) 42:499–511. doi: 10.1016/S0014-2921(98)00015-4

[20] Culter DM Reber SJ. Paying for health insurance: the trade-off between competition and adverse selection. Q J Econ. (1998) 113:433–66. doi: 10.1162/003355398555649

[21] Zhao SY Zang WB Yin QS. The welfare effect of medical insurance. J Econ Res. (2015) 8:130–45.

[22] Xiang H Du C Peng XB. Moral hazard of health insurance based on empirical evidence from compensation policy change. Insur Stud. (2020) 6:110–27. doi: 10.13497/j.cnki.is.2020.06.009

[23] Cohen A. Asymmetric information and learning: evidence from the automobile insurance market. Rev Econ Stat. (2005) 87:197–207. doi: 10.1162/0034653053970294

[24] Powell D Goldman D. Disentangling moral hazard and adverse selection in private health insurance. J Econ. (2021) 222:141–60. doi: 10.1016/J.JECONOM.2020.07.030, 33716385 PMC7945045

[25] Sheth K. Delivering health insurance through informal financial groups: evidence on moral hazard and adverse selection. Health Econ. (2021) 30:2185–99. doi: 10.1002/hec.4370, 34114717

[26] Lin C Hsu S. The effects of selection and moral hazard in additional health insurance in a universal healthcare system: evidence from Taiwan. Geneva Pap Risk Insur Issues Pract. (2025) 50:444–66. doi: 10.1057/s41288-024-00333-0

[27] Nguyen MT. Moral hazard and adverse selection in health insurances, evidence from a transitional economy. Singap Econ Rev. (2014) 59:1450011. doi: 10.1142/S0217590814500118

[28] Feng J Wang Z Song H. Self-selection and medical expenditure in Chinese health insurance system: evidence from informal employees’ participation. J Financ Res. (2018) 8:85–101.

[29] Abbring JH Heckman JJ Chiappori PA Pinquet J. Adverse selection and moral hazard in insurance: can dynamic data help to distinguish? J Eur Econ Assoc. (2003) 1:512–21. doi: 10.1162/154247603322391152

[30] Dionne G Michaud PC Dahchour M. Separating moral hazard from adverse selection and learning in automobile insurance: longitudinal evidence from France. J Eur Econ Assoc. (2013) 11:897–917. doi: 10.1111/jeea.12018

[31] Nghiem S Graves N. Selection bias and moral hazard in the Australian private health insurance market: evidence from the Queensland skin cancer database. Econ Anal Policy. (2019) 64:259–65. doi: 10.1016/j.eap.2019.09.008

[32] Franc C Perronnin M Pierre A. Supplemental health insurance and healthcare consumption-a dynamic approach to moral hazard. Health Econ. (2016) 25:1582–98. doi: 10.1002/hec.3271, 26468078

[33] Afoakwah C Byrnes J Scuffham P Nghiem S. Testing for selection bias and moral hazard in private health insurance: evidence from a mixed public-private health system. Health Econ. (2023) 32:3–24. doi: 10.1002/hec.4605, 36100982 PMC10087718

[34] Dong Y. How health insurance affects health care demand-a structural analysis of behavioral moral hazard and adverse selection. Econ Inq. (2013) 51:1324–44. doi: 10.1111/j.1465-7295.2012.00457.x

[35] Bardey D Buitrago G. Supplemental health insurance in the Colombian managed care system: adverse or advantageous selection? J Health Econ. (2017) 56:317–29. doi: 10.1016/j.jhealeco.2017.02.008, 29248058

[36] Yuan Z Sun YM Chen Z. Moral hazard in the commercial medical insurance in China. Insur Stud. (2014) 6:53–62. doi: 10.13497/j.cnki.is.2014.06.002

[37] Yin XJ Liu XH. Separate test of adverse selection and moral hazard in commercial medical insurance. Financ Econ. (2020) 7:33–41. doi: 10.19622/j.cnki.cn36-1005/f.2020.07.004

[38] Bajari P Dalton C Hong H Khwaja A. Moral hazard, adverse selection and health expenditures: a semiparametric analysis. Rand J Econ. (2014) 45:747–63. doi: 10.1111/1756-2171.12069

[39] Zhong XM Yang LM Lu JK. Testing adverse selection effect related to medical insurance. Financ Trade Econ. (2018) 39:118–30. doi: 10.19795/j.cnki.cn11-1166/f.2018.10.009

[40] Wang EN Zhu ML. The effect of moral hazard on basic medical insurance fund in the reform of basic medical insurance. Q J Financ. (2025) 19:106–26.

[41] Hartog J Ferrer-i-Carbonell A Jonker N. Linking measured risk aversion to individual characteristics. Kyklos. (2002) 55:3–26. doi: 10.1111/1467-6435.00175

[42] Nyman JA Koc C Dowd BE Mccreedy E Trenz HM. Decomposition of moral hazard. J Health Econ. (2018) 57:168–78. doi: 10.1016/j.jhealeco.2017.12.003, 29275240

[43] Rosenblatt M. Remarks on some nonparametric estimates of a density function. Ann Math Stat. (1956) 27:832–7. doi: 10.2307/2237390

[44] Silverman BW. Density estimation for statistics and data analysis. London: Chapman and Hall (1986).

[45] Bowman AW Adelchi A. Applied smoothing techniques for data analysis. New York: Oxford University Press (1997).

[46] Wik M Aragie KT Bergland O Holden ST. On the measurement of risk aversion from experimental data. Appl Econ. (2004) 36:2443–51. doi: 10.1080/0003684042000280580

[47] Jianakoplos NA Bernasek A. Are women more risk averse? Econ Inq. (1998) 36:620–30. doi: 10.1111/j.1465-7295.1998.tb01740.x

[48] Gabel JR Redisch MA. Alternative physician payment methods: incentives, efficiency and national health insurance. Milbank Mem Fund Q Health Soc. (1979) 57:38–59. doi: 10.2307/3349747, 253197

[49] National Healthcare Security Administration. Notice on the issuance of the three-year action plan for the DRG/DIP payment reform. (2021). Available online at: https://www.nhsa.gov.cn/art/2021/11/26/art_37_7406.html?from=timeline (Accessed November 26, 2021).

